# UK funding agency launches digital health hubs: a new catalyst for change?

**DOI:** 10.1038/s41746-023-00990-w

**Published:** 2024-01-06

**Authors:** A. Godfrey, D. Powell

**Affiliations:** 1 Department of Computer and Information Sciences, Northumbria University, Newcastle, UK; 2 Faculty of Health Sciences & Sport, University of Stirling, Stirling, Scotland, UK

**Keywords:** Funding

In 2021, the UK government’s life sciences set out an ambitious strategy and vision for investment, innovation, and collaborative practice^1^ to respond to the growing “silent pandemics” facing the UK, such as diabetes, obesity, and dementia. Underpinning that vision and aligned to recently launched workforce plans^2^ in England is the recognition that health systems must embrace new “ways of working,” which include the scaled adoption of digital technologies, AI, and a preventative approach to healthcare.

The UK-based Engineering and Physical Sciences Research Council (EPSRC) is the main funding body that supports innovative ideas for transformative technologies in health (among other sectors). To ensure that UK’s National Health Service is fit for purpose and meeting contemporary challenges, the EPSRC is aiding the development of technologies that include biopharmaceuticals, medical technology, genomics, diagnostics, and digital health approaches^3^. The EPSRC strategy is not limited to national priorities, aiming to ensure global impact tackling social, economic, and environmental challenges concurrently^4^.

That strategy led to the June 2023 EPSRC announcement that five large-scale multidisciplinary digital health hubs will be created, each with a dedicated focus area (Fig. 1). These will be primarily funded by the agency through a program centered on research and partnership for health technologies^5^. Each hub will be aimed at supporting partnerships across a wide research and health landscape, ensuring complementary expertise to co-deliver on objectives. The creation of each hub is to tackle three key health challenges described by the EPSRC: (i) improving population health and prevention, (ii) transforming prediction and early diagnosis, and (iii) discovering and accelerating the development of new interventions. Of note is the requirement for each hub to undertake public and patient involvement and engagement and partnership working to ensure health technology outcomes are designed to benefit end users and industry while also having maximum impact in the health sector.

**Fig. 1:**
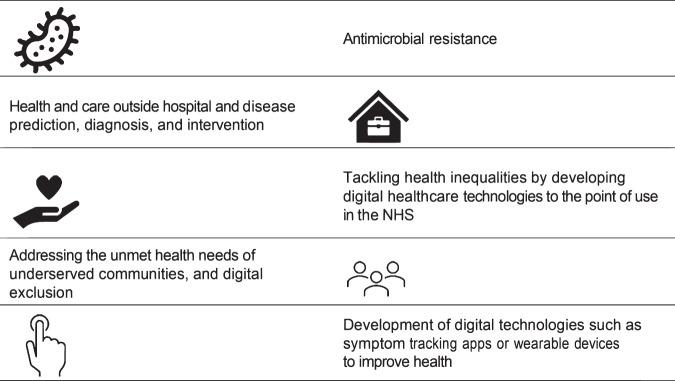
Recently, five digital health hubs were launched within the UK (https://www.ukri.org/news/innovative-healthcare-tech-could-transform-medicaltreatments). Those hubs are defined across different topics of healthcare priority asdecribed here.

## Global perspectives on digital healthcare

The UK’s strategy in the creation of these hubs is like digital health initiatives seen elsewhere, which bring together research and development in technologies, provision of training and resources, and promotion of collaboration between different stakeholders. The US National Institutes of Health funds the National Center for Advancing Translational Sciences and the National Human Genome Research Institute. In Europe, the European Union’s European Institute of Innovation and Technology (EIT Health) has a similar remit. Interestingly, a 2016 partnership-driven Innovation in Singapore set out the “*three beyond’s*” to encapsulate a similar strategy: namely “beyond health care to health, beyond hospital to community, and beyond quality to value.” This UK strategy and the international examples show a growing momentum to enable large-scale digital health innovation, and through this to drive global impact.

## Considerations for the future

Digital innovation and new ways of working are required to combat the significant challenges to global healthcare delivery. With significant geographical variations in healthcare outcomes in the UK, a locally led and nationally supported ecosystem for digital health development provides an important first step to provide solutions that are fit for purpose for all communities. Moving forward, the key yardstick of success will be the translation of hub ideas into tangible impact for local populations and communities, for national healthcare system development, and further impact beyond borders. For example, wearable technology has transformative potential but integration of this with existing infrastructure is challenging^6,7^. While there are many challenges facing health systems globally, this announcement of investment provides a much-needed catalyst and opportunity to realizing the potential of digital medicine.
